# Harnessing click detectors for the genuine characterization of light states

**DOI:** 10.1038/srep19489

**Published:** 2016-01-14

**Authors:** René Heilmann, Jan Sperling, Armando Perez-Leija, Markus Gräfe, Matthias Heinrich, Stefan Nolte, Werner Vogel, Alexander Szameit

**Affiliations:** 1 Institute of Applied Physics, Abbe Center of Photonics, Friedrich-Schiller-Universität Jena, Albert-EInstein-Straße 15, 07745 Jena, Germany; 2 Arbeitsgruppe Quantenoptik, Institute für Physik, Universität Rostock, D-18051 Rostock, Germany; 3 Fraunhofer Institute for Applied Optics and Precision Engineering, Albert-Einstein-Straße 707745 Jena, Germany

## Abstract

The key requirement for harnessing the quantum properties of light is the capability to detect and count individual photons. Of particular interest are photon-number-resolving detectors, which allow one to determine whether a state of light is classical or genuinely quantum. Existing schemes for addressing this challenge rely on a proportional conversion of photons to electrons. As such, they are capable of correctly characterizing small photon fluxes, yet are limited by uncertainties in the conversion rate. In this work, we employ a divide-and-conquer approach to infallibly discerning non-classicality of states of light. This is achieved by transforming the incident fields into uniform spatial distributions that readily lend themselves for characterization by standard on-off detectors. Since the exact statistics of the light stream in multiplexed on-off detectors are click statistics, our technique is freely scalable to accommodate–in principle–arbitrarily large photon fluxes. Our experiments pave the way towards genuine integrated photon-number-resolving detection for advanced on-chip photonic quantum networks.

Quantum information science is at the cutting edge of modern physics and technology. In this context, perhaps the most ambitious goal is to realize scalable quantum information processing and computing based exclusively on linear optical configurations and photon-counting devices^1^,^2^,^3^. Notably, any such optical quantum-computing scheme hinges on the ability to detect and manipulate the states of light at the single-photon level: Quantum cryptography, entanglement swapping, and quantum teleportation, to name a few, would clearly be impossible without reliable single-photon-counting devices^4^,^5^,^6^,^7^,^8^,^9^,^10^. Moreover, exact photon counts provide access to genuine photon number statistics, and in turn are the principal means of reliably establishing the non-classicality of any type of light field^11^,^12^,^13^,^14^,^15^,^16^,^17^,^18^. Another potential application of photon-number-resolving detectors (PNRs) was recently highlighted in the context of coherent optical communications^19^, where they enable coherent optical communications with a performance superior to the standard quantum limit, even in the high mean photon number regime. To this date, the perhaps most noticeable scheme for PNRs is based on superconducting nanowires^20^. Yet, on many occasions, cryogenic measurements may be impractical, or the incident photon flux may exceed the capacity of the system. Evidently, a fundamentally different approach will be required to reconcile the demands for high speed, low noise, and maximized quantum efficiency with the ever increasing count rates required by modern technologies^21^,^22^,^23^,^24^,^25^.

Existing schemes for measurements at the single-photon level employ on-off detectors, e.g. avalanche photodiodes (APDs)^11^, and as such are inherently limited by the so-called dead time. When an APD is triggered, it typically remains “blind” for several nanoseconds thereafter, and as a result, succeeding photons impinging on the detector cannot be registered^16^. In addition to being detrimental to the overall detection efficiency, this effect may corrupt the very state of light one strives to characterize. Moreover, this saturation effect also introduces undesired correlations to the count sequences^26^.

In contrast to PNRs, on-off detectors deliver well-defined “clicks” upon excitations with any non-zero number of photons^27^. Consequently, the by far most accessible quantum-optical measurements are click-counting statistics, instead of actual photon counts^28^. The question naturally arises as to whether it is possible to circumvent the limitations of on-off detectors, and to exploit these robust and widely available components to accurately characterize multiphoton states of light.

In this work, we propose, implement, and characterize a photon counting device based on a multiplexed array of on-off detectors^29^,^30^. In our arrangement, the discrete evolution dynamics of the incident light field is manipulated so as to spatially distribute the photons uniformly between the individual channels. Crucially, the click-counts obtained from these types of multiplexed sensors are used to reliably probe the non-classicality of arbitrary light fields^31^. Moreover, such click-counting statistics converge to the actual photon-counting statistics as the number of on-off detectors is increased 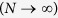
31.

Let us first consider a stream of single-photon states being routed through a uniform 1-to-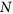 multiplexer and onto an array of avalanche photo diodes (APDs) (see Fig. 1, top). In our approach, this is achieved by cascading 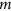 stages of 50/50 beam splitters, yielding 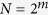. Under these premises, every single-photon will have a probability of 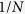 to be detected in one of the 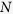 channels. Due to the spatially extended wave function, any two incoming photons are likely to be found in different outputs with a probability of 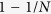: When one photon is detected, the global probability of registering the next photon in any of the remaining APDs is 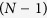 times greater than in the same one. This remains true even if the two photons enter the system simultaneously. In this manner, the fidelity of the device is expected to improve with the number of output ports, and is even independent of the type of input state^31^.

To experimentally demonstrate the functionality of our approach, we realized a discrete network of integrated 50/50 beam splitters cascaded in 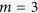 steps, yielding a total of 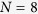 output channels. These photonic structures were implemented in fused silica glass by means of the femtosecond laser writing technique^32^,^33^, see Methods. As input states we consider two limiting cases: i) Low-intensity laser light and ii) heralded single photons from a spontaneous parametric down conversion source. Note that the actual photon statistic describing laser light exhibits substantial temporally fluctuating bunching of photons. As a result, it represents a perfect test case to demonstrate the capability of our setup.

In the multiplexer, pure coherent states 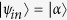 are split into eight spatially separated coherent states of equal amplitude, i.e. 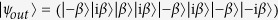, with 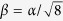. Consequently, a perfect photon counting characterization should yield a Poissonian photon number distribution. When one instead considers the click coincidences, it can be analytically shown that the resulting click-counting statistics have to obey a binomial distribution^31^. Note that in case of a sub- (or super-) Poissonian photon number distribution, it likewise follows that the click statistics are sub- (or super-) binomial, respectively (see for instance Fig. 2).

In general, this behavior with respect to 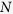 on-off detectors is mathematically described by the expectation value 
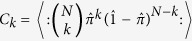
. Here, the normal ordering for the bosonic operators^34^ is indicated by the symbol 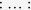, the number of APDs that click in a certain time window is 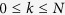, and the operator 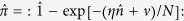 includes the photon number operator 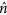 and accounts for a realistic detector with quantum efficiency 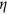 and dark counts 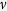. In this expression, the exponential operator corresponds to the projector of the vacuum operator, and as expectation value yields the probability of zero clicks^34^.

In order to quantify the binomial character of the click-counting statistics, we employ the parameter


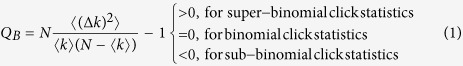


where 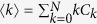 and 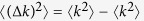
represent the average number of clicks and the variance thereof, respectively^35^. For an ideal coherent state, the mean and the variance of the click-counting statistics are analytically calculated as 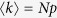 and 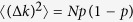, respectively, with 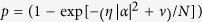. When substituting these two expressions into Eq. (1) we find that 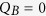 regardless of the quantum efficiency 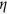 and the dark count rate 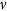. Accordingly, it is a sufficient criterion to differentiate between classical and non-classical light: while super-binomial click statistics such as those of realistic laser light are characterized by 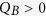, genuine quantum entities, such as Fock states, necessarily features 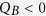. Note that in order for this parameter to be meaningful, the ensemble has to include 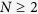 on-off detectors, otherwise measurements of any input state would produce 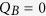
36,37.

In a first set of experiments, we measured the absolute number of click coincidences 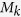 and the relative frequencies 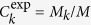 for attenuated laser light, where 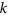 again represents the number of clicks within a time window of 10 ns and 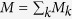 is the total number of time windows. Further details on our setup are given in the Methods section. From these measurements, we extract a positive value 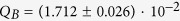, confirming that the click statistics is indeed super-binomial as expected for a classical light source. Beyond the 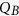 parameter, which relies solely on second-order correlations, one can also employ the higher-order correlations contained within the matrix of moments to identify non-classical behavior (see Supplementary Information).

In our second set of experiments, we used our device to characterize the fidelity of a heralded single-photon source based on spontaneous parametric down conversion. As in the previous case, the input state 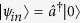 is spatially distributed and thus transformed according to the expression 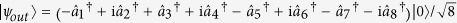, where 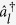 represents the bosonic creation operators of the 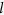
^th^ waveguide mode. The high fidelity of our device is confirmed by the homogeneity of the single photon number output distribution with an average of 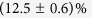
(see Fig. 1, bottom). Measurement data clearly demonstrates the non-classicality of the input state, with 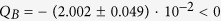. At this point we emphasize that the 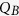 parameter corresponding to pure single photon states can be estimated analytically by


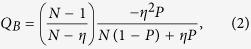


where 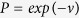 (see Supplementary Information). For the ideal case when a pure single-photon Fock state is detected by a perfect photon-counting device with detection efficiency 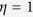 and 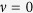, Eq. (2) yields 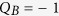. In our experiments, the number of photons, which determines the number of clicks per time bin, is naturally limited by the brightness of the source. As a result, experimental 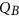 parameters are expected to lie in the interval 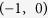.

Additionally, we analyzed the dependence of the fidelity of our device for different source brightness levels. To this end, we determined the 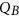 parameter for classical laser light with different attenuation ratios, as well as for single-photon states at different count rates. Figure 3 illustrates that our scheme allows for a clear distinction between classical and non-classical behavior. Note that, as the incident intensity decreases, the chance of multiple photons entering the device in any given time slot gradually converges to zero, as does the corresponding 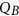 parameter. Whereas the sign of 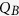 remains well defined throughout this process, the uncertainty determined by the experimental conditions [see Methods] eventually exceeds the absolute value. Nevertheless, standard quantum sources routinely feature count rates well above this limit, placing them firmly within the window of confidence of our characterization scheme.

In our experiments, the number of photons is naturally limited by the brightness of the source, and it determines the number of clicks per time bin. As a result, the actual detection efficiency 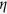 of the system is directly related to the measured click statistics, 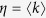. Therefore, by plugging our experimental 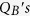 into Eq. (2), we find a negligible noise count rate of 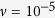, as expected from a heralded photon source (see Fig. 4).

In conclusion, we have introduced a new paradigm for integrated photon-number resolved measurements based on distributed sensing with multiplexed arrays of conventional on-off detectors. In this divide-and-conquer approach, the limiting factor of detector dead time is overcome by transforming the incident fields into extended uniform distributions. As such, coincidences in the same measurement channel are reliably suppressed even for highly multi-photon input states. Measuring the click statistics of the detector ensemble therefore provides the means to determine the actual photon-counting statistics. Consequently, our technique can in principle be scaled to allow for arbitrarily high numbers of incident photons, irrespective of the dead time of the individual detectors used. Our experiments pave the way towards genuine integrated photon-number-resolving detectors for advanced on-chip photonic quantum networks.

## Methods

### Fabrication & characterization

The multiplexing waveguide network was fabricated by means of the direct femtosecond laser inscription in fused silica glass^30^,^31^. It is designed to match the technical standards of the attached V-groove fiber array with 127 μm pitch which collects the photon outcome and feeds it into single-photon click-detectors. In order to inject weak laser light, different neutral density (ND) filters were placed into the beam of a laser diode emitting at 808 nm. The attenuated light was coupled into a single mode (SM) polarization maintaining (PM) fiber attached to the input wave guide of the multiplexer with a maximum photon flux of 2 million per second. For the other set of experiments, single photons of 815 nm were generated by spontaneous parametric down conversion (SPDC) in a BiB_3_O_6_ crystal and coupled into two SM and PM fibers. One of the fibers was directly connected to a single-photon detector to provide a herald for the other twin photon, which was delivered directly to the device’s injection site. The heralding efficiency before the glass chip was 18% yielding 65 thousand coincidences per second. By using the second photon as trigger, we can suppress any noticeable influence of dark counts. For reasons of experimental convenience, the signal photon flux was again controlled by an appropriate choice of ND filters in order to change the vacuum component of the state.

### Measurement methods & errors

The photon clicks by our APDs were collected by a time tagging card capable to handle up to 16 detectors at the same time with a timing resolution of 168 ps. The coincidence time window, wherein two or more clicks are interpreted as a joint event, was set to 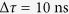 for all measurements. In turn, the number of measurements for attenuated laser light was determined by 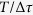, where 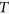 is the overall recording time. In contrast, the number of quantum measurements was determined by the photon flux of the trigger photons, and therefore remains independent of the coincidence time window. In both cases, the overall measurement time was chosen in a way to collect at least 100 million non-zero click events, ranging from 44 seconds up to more than 60 hours. The employed APDs had a dark-count rate of less than per second, a timing jitter of 350 ps and a dead time of 20 ns.

**Figure 1:**
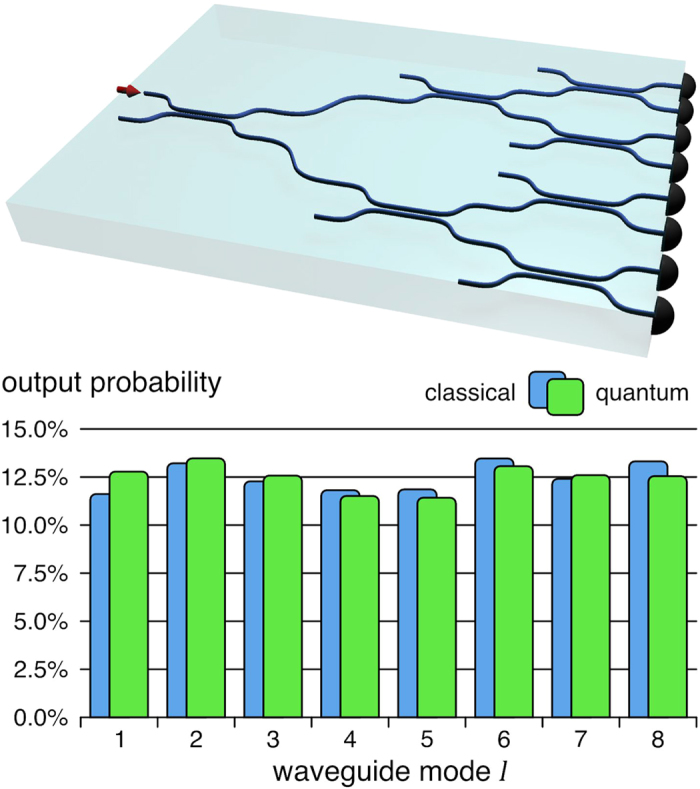
Top: Sketch of the 1-to-8 optical integrated multiplexer consisting of three beam splitting stages. The output fields are fed into APDs. Bottom: Output click statistic of the above multiplexer for classical attenuated laser light (blue), as well as single-photon Fock-states (green). In the experiment, both input states yield a flat uniform output statistic provided by the high quality of the optical-integrated device.

**Figure 2:**
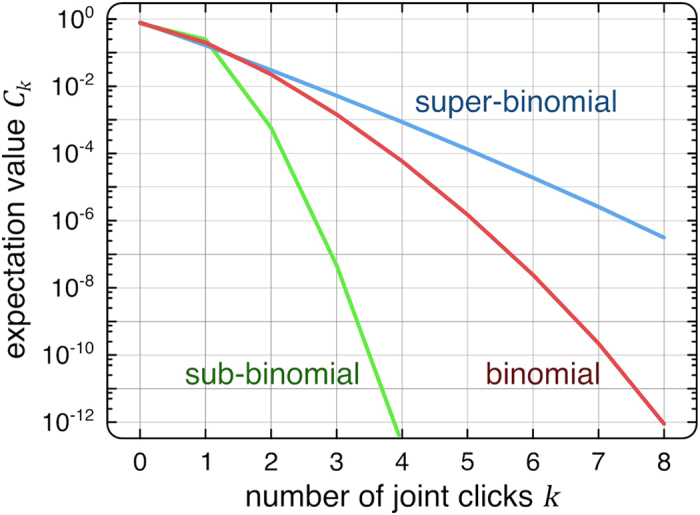
Theoretical click-counting statistics for three different photon number distributions at an average number of clicks of 0.25 per measurement. The resulting 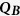 values are 0 for binomial, −0.22 for sub-binomial, and +0.22 for super-binomial click statistics.

The accuracy of the calculated *Q*_*B*_ parameter is chiefly dependent on the number of measurements. In an ideal set of data, the frequencies *C*_*k*_ would be distributed according to the graphs shown in Fig. 1. If a measurement is terminated before the highest number of joint clicks yields a non-zero quantity, it cannot perfectly match the (sub- or super-) binomial distribution. Consequently, the extracted *Q*_*B*_ parameter is subject to a systematic uncertainty. A second source of errors results from imperfections in the splitting ratio of the multiplexing device, corresponding to small deviations from the ideal case of homogeneously distributed outputs (see Fig. 2). This also influences the coincidence click statistics and, hence, the error for the *Q*_*B*_ parameter. In turn, the resulting overall range of error defines a certain minimum count rate.

**Figure 3:**
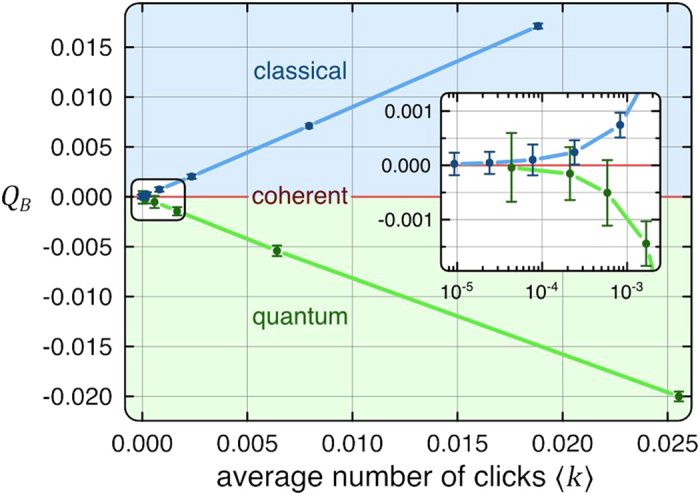
Experimentally obtained 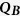 for two different click statistics versus a variation of the average click number 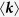. This was realized by using ND filters in the signal path. The inset shows a semilogarithmic section for the smallest achieved 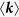
s. An attenuated laser light (blue curve) is always accompanied by a positive 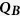 (super-binomial), whereas our single photon source (green curve) constantly shows a negative 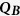 (sub-binomial). In both cases, a coherent state (binomial photon number distribution) is covered within the error-bars at very low count rates.

We would like to emphasize that our method is capable of reliably identifying the signature of the input state, whether classical or quantum (see Fig. 3), without the need for any corrections or post processing of raw data.

## Additional Information

**How to cite this article**: Heilmann, R. *et al*. Harnessing click detectors for the genuine characterization of light states. *Sci. Rep.* **6**, 19489; doi: 10.1038/srep19489 (2016).

## Supplementary Material

Supplementary Information

## Figures and Tables

**Figure 4:**
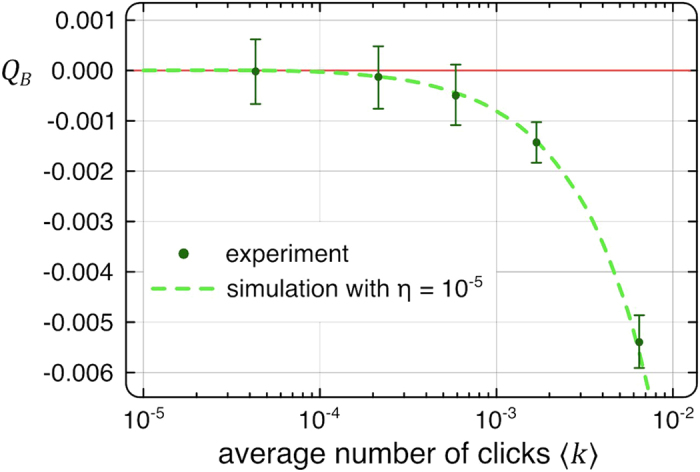
Comparison between the experimentally obtained 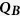
 **parameters of a heralded single photon source and our analytical expression Eq. (2) for a different average click number** 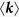. The average click numbers 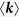 are extracted from the experimental click statistics, (horizontal axis in Fig. (3)). This in turn provides the actual detection efficiency 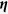 of our system. The measured values of 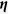, in combination with the experimental 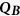, yield a negligible noise count rate of 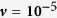, as expected from a heralded photon source.
