# Supplementary material for: Harnessing click detectors for the genuine characterization of light states
Source: Sci Rep. 2016 Jan 14;6:19489. doi: 10.1038/srep19489 (PMC4725864; doi:10.1038/srep19489)
Supplement: Supplementary Information [file srep19489-s1.docx]

**Supplementary Information document**

**for**

**Harnessing click detectors**

**for the genuine characterization of light states**

René Heilmann,^1,†^ Jan Sperling,^1,†^ Armando Perez-Leija,^1,†^ Markus Gräfe,^1^ Matthias Heinrich,^1^ Stefan Nolte,^1^ Werner Vogel^2^ and Alexander Szameit^1,*^

^1^ Institute of Applied Physics, Abbe Center of Photonics, Friedrich-Schiller-Universität Jena, Max-Wien-Platz 1, 07743 Jena, Germany

^2^ Arbeitsgruppe Quantenoptik, Institute für Physik, Universität Rostock, D-18051 Rostock, Germany

^†^ these authors contributed equally

^*^ To whom correspondence should be addressed. E-mail: alexander.szameit@uni-jena.de

**Analytical** $\mathbf{Q}_{\mathbf{B}}$ **parameter for single photon Fock states**

In order to obtain an analytical expression for the $Q_{B}$ parameter corresponding to a Fock state, we consider the density operator of a coherent state

$\left. \left| \alpha\right. \right\rangle\left\langle\left. \alpha\right|=\exp\left( -{|\alpha|}^{2} \right)\exp\left( \alpha\hat{a}^{\dagger} \right) \right.\left. \left| \alpha\right. \right\rangle\left. \left\langle\alpha\right. \right|\exp\left( \alpha^{*}\hat{a} \right).$ (S1)

Using this expression, we can cast a Fock state $\left. \left| m \right. \right\rangle\left. \left\langle m \right. \right|$ as follows

$\frac{1}{m!}\partial_{\alpha}^{m}\partial_{\alpha^{*}}^{m}\left[ \left. \left| \alpha\right. \right\rangle\left. \left\langle\alpha\right. \right| \right]|_{\alpha=0}=\frac{1}{m!}\hat{a}^{\dagger m}\left. \left| 0 \right. \right\rangle\left. \left\langle0 \right. \right|\hat{a}^{m}=\left. \left| m \right. \right\rangle\left. \left\langle m \right. \right|.$ (S2)

Eq. (S2) is particularly useful, because quantum mechanical expectation values for $\left. \left| m \right. \right\rangle$ photon states can be computed in terms of derivatives as follows

$\left. \left\langle m \right. \right|\hat{L}\left. \left| m \right. \right\rangle=\frac{1}{m!}\partial_{\alpha}^{m}\partial_{\alpha^{*}}^{m}\left[ \exp\left( -{|\alpha|}^{2} \right)\left. \left\langle\alpha\right. \right|\hat{L}\left. \left| \alpha\right. \right\rangle\right]|_{\alpha=0}.$ (S3)

For the special case when $\left. \left| m \right. \right\rangle=\left. \left| 1 \right. \right\rangle$, and assuming an operator $\hat{L}$ which only depends on the number operator $\hat{n}$, one can see that Eq. (S3) yields

$\partial_{\alpha}\partial_{\alpha^{*}}\left[ \exp\left( -{|\alpha|}^{2} \right)L\left( \alpha\right) \right]|_{\alpha=0}=L\left( 0 \right)-L^{'}\left( 0 \right),$ (S4)

with $L^{'}\left( x \right)=\partial_{x}L\left( x \right)$. From the click counting statistics of a coherent state [S1]

$c_{k}=\left( \begin{aligned} N \\ k \end{aligned} \right)\left[ \exp\left( -\left( \eta\frac{{|\alpha|}^{2}}{N}+\nu\right) \right) \right]^{N-k}\left[ 1-\exp\left( -\left( \eta\frac{{|\alpha|}^{2}}{N}+\nu\right) \right) \right]^{k},$ (S5)

we obtain the first and second factorial moments

$\left\langle k \right\rangle=N\left[ 1-\exp\left( -\left( \eta\frac{{|\alpha|}^{2}}{N}+\nu\right) \right) \right]$, (S6)

and

$\left\langle k\left( k-1 \right) \right\rangle=N\left( N-1 \right)\left[ 1-\exp\left( -\left( \eta\frac{{|\alpha|}^{2}}{N}+\nu\right) \right) \right]^{2}.$ (S7)

Note that $\eta$ and $\nu$ represent the detection efficiency and the noise count rate. Using Eq. (S4) in combination with Eq. (S5), (S6), and (S7), we obtain the click-counting statistics for single photon Fock states

$c_{k}=\left( \begin{aligned} N \\ k \end{aligned} \right)P^{N-k}\left( 1-P \right)^{k}\left[ 1-\eta+\frac{k\eta}{N\left( 1-P \right)} \right].$ (S8)

And the first and second moments

$\left\langle k \right\rangle=N\left( 1-P \right)+\eta P$, (S9)

and

$\left\langle k\left( k-1 \right) \right\rangle=\left( N-1 \right)\left( 1-P \right)\left[ N\left( 1-P \right)+2\eta P \right].$ (S10)

Here we have defined $P=\exp\left( -\nu\right)$. Using these expressions we calculate the corresponding $Q_{B}$ parameter for single photon Fock states

$Q_{B}=\left( \frac{N-1}{N-\eta} \right)\frac{-\eta^{2}P}{N\left( 1-P \right)+\eta P}.$ (S11)

For the ideal case when the efficiency $\eta=1$ and $\nu=0$, Eq. (11) reduces to $-1$. For a realistic photon source producing mixed states of the form $\left. \eta_{1}\left| 0 \right. \right\rangle\left. \left\langle0 \right. \right|+\left( 1-\eta_{1} \right)\left. \left| 1 \right. \right\rangle\left. \left\langle1 \right. \right|$, one can show that the $Q_{B}$ parameter is given by Eq. (S11) provided we exchange $\eta\to{\eta\eta}_{1}$.

**High-order correlations**

In order to analyze high order correlations of the light fields under study, we use the approach recently introduced in [S1]. Along these lines, we compute the matrix of moments $\left( MoM \right)$ directly from the experimental click-counting statistics $C_{k}^{\exp}$. This is done using the expression $\left( MoM \right)_{m,n}=\left( \left\langle:\hat{\pi}^{m+n}: \right\rangle\right)_{m,n}$. Here,

$$\left\langle:\hat{\pi}^{m+n}: \right\rangle=\frac{\left( N-\left[ m+n \right] \right)!}{N!}\sum_{k=0}^{N} k\left( k-1 \right)\ldots\left( k-\left[ m+n \right]+1 \right)C_{k}^{\exp}, (S12)$$

with the indices $m,n$ ranging from $0$ to $\left\lfloor N/2 \right\rfloor$, where $\left\lfloor\ldots\right\rfloor$ represents the floor function. It can be shown that the principal minors of the $MoM$ can be expressed in terms of the $Q_{B}$ parameter, and as a result for classical states the associated $MoM$ is expected to be non-negative. The semi-definiteness of the $MoM$ can be established through the analysis of corresponding eigenvectors and eigenvalues. In other words, the $MoM$ will be positive definite if all its eigenvalues are positive. Therefore, if we find at least one negative eigenvalue, we can unequivocally conclude that the light field being characterized is non-classical. This condition is mathematically described by

$$0\leq\vec{f}^{\dagger}\left( MoM \right)\vec{f}=\left\langle:\hat{f}^{\dagger}\hat{f}: \right\rangle=\sum_{m,m^{'}}^{\left\lfloor N/2 \right\rfloor} f_{m}^{*}f_{m^{'}}\left\langle:\hat{\pi}^{m+m^{'}}: \right\rangle, (S13)$$

where we have defined the operators $\hat{f}=f_{0}+f_{1}\hat{\pi}+\ldots+f_{\left\lfloor D/2 \right\rfloor}\hat{\pi}^{\left\lfloor N/2 \right\rfloor}$ and $\vec{f}=\left( f_{0},\cdots,f_{\left\lfloor N/2 \right\rfloor} \right)$ represents the eigenvectors of $MoM$. For a classical source, the moments can be calculated analytically using the expression $\left\langle:\hat{\pi}^{m}: \right\rangle=p^{m}=\left( 1-\exp\left[ -\left( \eta\left| \alpha\right|^{2}+\nu\right)/N \right] \right)^{m}$. This indicates that the $MoM$ of an ideal coherent state exhibits one negative eigenvalue given by $\left( 1-p^{2\left\lfloor N/2 \right\rfloor} \right)/\left( 1-p^{2} \right)$, and a total of $\left\lfloor N/2 \right\rfloor$ zero eigenvalues for $N>2$.

Here, we consider a $1$-to-$8$ multiplexer device, which corresponds to a $5\times5$ $MoM$:

$$MoM=\left( \begin{matrix} \left\langle:\hat{\pi}^{0}: \right\rangle& \cdots& \left\langle:\hat{\pi}^{4}: \right\rangle\\ \vdots& \ddots& \vdots\\ \left\langle:\hat{\pi}^{4}: \right\rangle& \cdots& \left\langle:\hat{\pi}^{8}: \right\rangle\end{matrix} \right) . (S14)$$

Using the measured $C_{k}^{\exp}$, we numerically obtain the eigenvalues and eigenvectors. The results of this analysis for experimental data obtained over 83.2 s (corresponding to $M=8.32\cdot{10}^{9}$ windows) are shown in Tables SI and SII for the classical and quantum states, respectively, whereas Figure 3 of the main manuscript shows a graphical representation of these quantities.

| Eigenvalues | $\left\langle:\hat{f}^{\dagger}\hat{f}: \right\rangle$ | Standard Error | $\sum$ |
| --- | --- | --- | --- |
| 1 | $\left( 1\times{10}^{00} \right)\pm\left( 3\times{10}^{-10} \right)$ | $3.2936\times{10}^{-10}$ | $3.03619\times{10}^{9}$ |
| 2 | $\left( 1.40231\times{10}^{-06} \right)\pm\left( 3\times{10}^{-09} \right)$ | $3.88066\times{10}^{-9}$ | $361.359$ |
| 3 | $\left( 4.3309\times{10}^{-13} \right)\pm\left( 3\times{10}^{-12} \right)$ | $4.43711\times{10}^{-12}$ | 0.0976064 |
| 4 | $\left( -8.59786\times{10}^{-16} \right)\pm\left( 3\times{10}^{-15} \right)$ | $8.87048\times{10}^{-15}$ | 0.0969266 |
| 5 | $\left( -2.47982\times{10}^{-22} \right)\pm\left( 3\times{10}^{-22} \right)$ | $6.09801\times{10}^{-22}$ | 0.406661 |

Table SI: Correlation measurements for classical coherent light. As expected from a classical source no one of the eigenvalues is significantly negative.

| Eigenvalues | $\left\langle:\hat{f}^{\dagger}\hat{f}: \right\rangle$ | Standard Error | $\sum$ |
| --- | --- | --- | --- |
| 1 | $\left( 1.00001\times{10}^{00} \right)\pm\left( 3\times{10}^{-10} \right)$ | $7.77014\times{10}^{-9}$ | $1.287\times{10}^{8}$ |
| 2 | $\left( -1.38281\times{10}^{-05} \right)\pm\left( 3\times{10}^{-09} \right)$ | $1.6425\times{10}^{-8}$ | $841.894$ |
| 3 | $\left( -3.78528\times{10}^{-14} \right)\pm\left( 3\times{10}^{-12} \right)$ | $4.47567\times{10}^{-14}$ | 0.845747 |
| 4 | $\left( 2.78037\times{10}^{-21} \right)\pm\left( 3\times{10}^{-15} \right)$ | $3.29078\times{10}^{-21}$ | 0.844898 |
| 5 | $0\pm\left( 0\times{10}^{-22} \right)$ | 0 | $Indetermined$ |

Table SII: Correlation measurements for the single-photon Fock states.

The significance for these experimentally obtained eigenvalues can be quantified using the expression $\Sigma=\frac{\left| \left\langle:\hat{f}^{\dagger}\hat{f}: \right\rangle\right|}{\sigma\left( \left\langle:\hat{f}^{\dagger}\hat{f}: \right\rangle\right)}$, where $\sigma\left( \left\langle:\hat{f}^{\dagger}\hat{f}: \right\rangle\right)$ is the standard error of the mean $\left\langle:\hat{f}^{\dagger}\hat{f}: \right\rangle$.

Note that the two negative eigenvalues, which unexpectedly occur in the classical case, can be considered zero since their absolute values fall within the error margin. The values of $\Sigma$ for all measurements are shown in the fourth column of Table SI, illustrating that all eigenvalues are non-negative within the range of their significance.

A similar validity check was also applied for our measurements of single photon Fock-states (Table SII). As in the previous case, we calculate the significance of the deviation from the classical bound, i.e. zero. This is done using the second eigenvector

$$\hat{f}=\left( 0.00384503, -0.999993,-0.000251841,-5.430982775\times{10}^{-8},0 \right)$$

which determines the operator test. The obtained value of $\Sigma=841.894$ certifies that the state under consideration is indeed non-classical.

**References**

1. J. Sperling, W. Vogel, and G. S. Agarwal, Phys. Rev. A **88**, 043821 (2013)
